# The Interactive Effects of Dietary Fish Oil and Selenium Nanoparticles Increased Growth, Antioxidant Capacity, and Immune-Related Genes Transcription Level in *Penaeus vannamei* Reared in Hypersaline Water

**DOI:** 10.1155/anu/4165191

**Published:** 2025-08-28

**Authors:** Ali Ahmadi, Vahid Yavari, Mansour Torfi Mozanzadeh, Seyed Mohammad Mousavi, Preeta Kochanian, Ahmad Ghasemi

**Affiliations:** ^1^Department of Fisheries, Faculty of Marine Natural Resources, Khorramshahr University of Marine Science and Technology, Khorramshahr, Iran; ^2^South Iran Aquaculture Research Institute, Iranian Fisheries Science Institute (IFSRI), Agricultural Research Education and Extension Organization (AREEO), Ahwaz, Iran; ^3^Department of Biotechnology, Persian Gulf Research Institute, Persian Gulf University, Bushehr, Iran

**Keywords:** long-chain polyunsaturated fatty acids, nanoelements, osmotic stress, penaeid, selenium

## Abstract

A 60-day research was conducted to evaluate the influence of dietary fish oil (FO) and selenium nanoparticles (SeNPs) on performance of *Penaeus vannamei* juveniles (2.4 ± 0.0 g) reared in seawater (SW) or hypersaline (HS) water conditions. A 2 × 2 × 2 factorial research was designed with two FO levels, including 3% and 6%, two SeNPs dosages, including 0.4 and 0.8 mg/kg and two water salinity, including SW (35 g/L) and HS (50 g/L). Eight experimental groups were designed as follow: (1) FO_3_Se^0.4^SW (3% FO + 0.4 mg/kg SeNPs reared in SW), (2) FO_3_Se^0.8^SW (3% FO + 0.8 mg/kg SeNPs reared in SW), (3) FO_6_Se^0.4^SW (6% FO + 0.4 mg/kg SeNPs reared in SW), (4) FO_6_Se^0.8^SW (6% FO + 0.8 mg/kg SeNPs reared in SW), (5) FO_3_Se^0.4^HS (3% FO + 0.4 mg/kg SeNPs reared in HS), (6) FO_3_Se^0.8^HS (3% FO + 0.8 mg/kg SeNPs reared in HS), (7) FO_6_Se^0.4^HS (6% FO + 0.4 mg/kg SeNPs reared in HS), and (8) FO_6_Se^0.8^HS (6% FO + 0.8 mg/kg SeNPs reared in HS). Four hundred and eighty *P. vannamei* were randomly distributed into 24 250-L cylindrical fiberglass tanks. Each experimental group was replicated in three tanks (20 shrimp/tank). Shrimps were fed at 5% of their initial biomass three times daily. Water temperature and dissolved oxygen levels were maintained at 31.5 ± 1.2°C and 5.5 ± 1.0 mg/L, respectively. Shrimp in FO_6_Se^0.4^SW and FO_6_Se^0.8^SW had higher weight gain (WG) compared to those in FO_3_Se^0.8^SW and FO_3_Se^0.4^HS (*p* < 0.05). Rearing shrimp in HS water increased whole-body protein and ash contents but reduced moisture level. The amount of docosahexaenoic acid (DHA) in FO_3_Se^0.4^SW, FO_6_Se^0.4^SW, FO_6_Se^0.8^SW, and FO_6_Se^0.4^HS was relatively higher than the other treatments. The antioxidant activities, including glutathione peroxidase (GPx), superoxide dismutase (SOD), catalase (CAT), and glutathione (GSH) level decreased and malondialdehyde (MDA) content in hepatopancreas increased in shrimp reared in HS water. Finally, the transcription levels of insulin-like growth hormones and immune-related genes, including lectin (*lec*), peneidine 3 (*pen-3*), prophenoloxidase (*propo*), and lysozyme (*lyz*) increased by increasing dietary FO level of 6%. Additionally, these levels were modulated by the interaction of water salinity and dietary SeNPs and FO levels. Based on the findings of the current study, increasing the dietary FO level to 6% and incorporating a moderate level of SeNPs (0.4 mg/kg) were found to enhance *P. vannamei* tolerance to HS water.

## 1. Introduction

Total crustacean aquaculture production was 12.8 million tons of which white leg shrimp (*Penaeus vannamei*), with 6.8 million tonnes comprises 53% of the cultured crustacean production [1]. This species is the main farmed crustacean species in Iran. There are many hypersaline (HS) water resources in the south of Iran with great potential for culture of euryhaline species such as *P. vannamei*. Aquaculture in HS waters such as HS lakes, desalination brine, and saline groundwater, presents a promising approach for conservation of freshwater resources and also offering economic gains [2]. However, prosperity of this approach depends on careful consideration of challenges related to osmoregulation, species selection, and nutrients availability [2]. For instance, HS water poses physiological challenges for aquatic animals, requiring them to regulate salt balance by increasing water intake and salt excretion. Consequently, species selection by identifying and cultivating aquatic species naturally tolerant to HS water sources is crucial. Furthermore, the unique chemical composition of HS water can influence the availability of nutrients required for the growth, osmoregulation, and health conditions of aquatic organisms [2]. Taking into consideration that high water salinity may negatively affect growth and disease resistance in *P. vannamei*, using functional feed additives to increase stress tolerance presents a promising strategy for improving shrimp aquaculture productivity under such challenging conditions [3].

Findings alternative sources for marine derived ingredients, particularly fish meal (FM) and fish oil (FO), is vital for sustainability of aquafeed industry [4]. However, replacement of FM with alternative protein sources (APSs), especially plant proteins in aquafeeds was found to adversely affect their nutritional value due to the presence of antinutritional factors, lower digestibility, and reduced trace element (TE) bioavailability [5]. For instance, low bioavailability of TE in diet can result in delayed growth, oxidative stress, and reduced immunocompetence in farmed aquatic species [5]. Thus, dietary TE supplementation should be considered based on the farmed aquatic species, when a large part of dietary FM is replaced with APS to compensate such deficiencies.

Among various TE, selenium (Se) is a vital element because of its integral role in synthesis of selenoproteins, such as glutathione peroxidase (GPx) that provides cell protection against oxidative damages [6]. Dietary Se deficiency may lead to retarded growth, oxidative stress, and malfunction of immune system [7]. However, dietary extra Se could be poisonous for aquatic species by disrupting antioxidant defense system [7, 8]. Regarding crustacean species, several studies have suggested an optimal Se range of 0.44–0.68 mg/kg to enhance antioxidant capacity, immunocompetence, and stress resistance [9–13]. In this regard, Yu et al. [14] showed that dietary L-selenomethionine supplementation at 0.84 mg/kg significantly increased survival, growth, and antioxidant capacity in *P. vannamei* reared in low salinity (3 g/L) water, indicating an integral of Se to mitigate the osmotic stress. In other studies the optimal dietary Se level for *P. vannamei*, was reported to be between 0.2–0.4 mg/kg [15–17].

On the other hand, the Se molecular form markedly affects its effectiveness in various farmed aquatic species. Among various Se sources, Se nanoparticles (SeNPs) have many beneficial characteristics such as lower toxicity, more bioavailability, higher permeability, and slow-release delivery system that promotes Se absorption [18]. In this context, Ghaffarizadeh et al. [17] showed that SeNPs (0.38 mg/kg) supplementation improved growth and feed efficiency, increased antioxidant capacity, and lowered lipid peroxidation levels in HP.

Along with Se, dietary long-chain polyunsaturated fatty acids (LC-PUFAs) also have pivotal role in growth, reproduction, stress resistance, and welfare of crustaceans [19]. FO is the main source of LC-PUFA in aquafeeds. In *P. vannamei* feed formulation, a minimum LC-PUFA range between 0.3%–0.5% (diet weight basis) is required, including 0.2% eicosapentaenoic acid (EPA) and 0.1%–0.3% docosahexaenoic acid (DHA) [20]. Dietary optimal level of LC-PUFA not only improved growth and feed efficiency but also it was found to enhance osmotic stress tolerance in *P. vannamei* [ 21–23]. It has been postulated that LC-PUFA may regulate ion balance through two mechanisms: by altering cell membrane fluidity and influencing permeability, and by acting as substrates for eicosanoid production, which regulates ion metabolism across the gill membrane [24]. In this context, Palacios et al. [21] reported that a medium amount of dietary LC-PUFA resulted in higher survival rates during osmotic stress challenge (10 g/L for 3 h) by modifying gill fatty acid (FA) composition, increasing gill area, elevating Na^+^/K^+^-ATPase pump, and carbonic anhydrase activities. The amount of dietary FO in the commercial diets used in shrimp culture in the south of Iran is about 3% [17] and the main source of FO is from anchovies (Engraulidae) and sardines (Clupeidae). However, due to impacts of climate change, reduced water exchange (commonly implemented to mitigate the risk of pathogen entry into the earthen culture ponds), resulting in rising water salinity, particularly in the shrimp coastal farms of southern Iran, the proportion of FO in the diet, as the main source of LC-PUFA, requires reassessment.

Studies on the interaction of dietary Se and LC-PUFA level in cultured aquatic species is scarce. In a study, Tseng et al. [25] investigated the impact of dietary hydroxy-selenomethionine supplementation on gilthead seabream (*Sparus aurata*), and showed that increment of dietary Se (0.79 mg Se/kg diet) increased Se retention in tissues, elevated lipid content, and enhanced retention and synthesis of n − 3 LC-PUFA, particularly DHA. In addition, high Se levels increased cortisol response to stress.

In the present study, the interactive effects of two levels of dietary FO (3% and 6%) as a source of LC-PUFA and two levels of SeNPs (0.4 and 0.8 mg/kg) were evaluated on growth, body composition, FA profile, antioxidant capacity, and immune-related genes mRNA transcription level in *P. vannamei* reared in seawater (35 g/L, SW) and HS water (50 g/L).

## 2. Materials and Methods

### 2.1. Experimental Design and Feeds

A 2 × 2 × 2 factorial research was designed with two FO levels, including 3% and 6%, two SeNPs dosages, including 0.4 and 0.8 mg/kg, and two water salinity, including SW, 35 and HS, 50 g/L. Based on this design, eight experimental treatments were as follows: (1) FO_3_Se^0.4^SW (3% FO + 0.4 mg/kg SeNPs reared in SW), (2) FO_3_Se^0.8^SW (3% FO + 0.8 mg/kg SeNPs reared in SW), (3) FO_6_Se^0.4^SW (6% FO + 0.4 mg/kg SeNPs reared in SW), (4) FO_6_Se^0.8^SW (6% FO + 0.8 mg/kg SeNPs reared in SW), (5) FO_3_Se^0.4^HS (3% FO + 0.4 mg/kg SeNPs reared in HS), (6) FO_3_Se^0.8^HS (3% FO + 0.8 mg/kg SeNPs reared in HS), (7) FO_6_Se^0.4^HS (6% FO + 0.4 mg/kg SeNPs reared in HS), and (8) FO_6_Se^0.8^HS (6% FO + 0.8 mg/kg SeNPs reared in HS) (Table 1). The SeNPs concentration was chosen based on prior studies that examined Se supplementation in *P. vannamei* diet. The dietary FO level was selected at 3% (Ca. 0.6% LC-PUFA) to meet the optimal dietary LC-PUFA requirement for *P. vannamei* juveniles, as reported by Gonzalez-Felix et al. [20]. Similarly, SeNPs level of 0.4 mg/kg was selected based on findings of Ghaffarizadeh et al. [17] in *P. vannamei* juveniles. Additionally, higher dietary FO at 6% (Ca. 1.2% LC-PUFA) and SeNPs (0.8 mg/kg) were selected to potentially meet *P. vannamei* nutritional requirements at HS water. Bovine serum albumin-loaded-Se-N (30–45 nm particle size, spherical in shape, 99.95% purity, 3.89 g cm^3^ true density, aquaculture grade, Iranian Nanomaterials Pioneers, Mashhad, Iran) was used in this study. Each mL of the bovine serum albumin-loaded-SeNPs contained 1 mg of SeNPs. In summary, dry ingredients were mixed (20 min) and blended with oils (10 min). SeNPs was dissolved in water and added to the mixture. Eventually, gelatin was dissolved in warm water and added to the feed blend to prepare a soft dough. A kitchen meat grinder (2 mm) was used to pellet the dough. The pellets were fan dried (30°C, 48 h), packed, and stored at −20°C until use. The chemical composition of the ingredients and diets were analyzed following AOAC [26].

### 2.2. Husbandry Conditions

Juvenile shrimps were transferred from a private shrimp nursery center in Bushehr province, Iran. Upon arrival, shrimps were disinfected using formalin (100 mg/L for 30 s) and acclimated to the husbandry conditions over a 2 weeks period [27]. Four hundred and eighty *P. vannamei* (2.4 ± 0.0 g, mean ± SE) were randomly distributed among 24 250-L cylindrical fiberglass tanks. Shrimps were first bulk weighed (20 animal/tank) and then stocked in the experimental tanks. Each experimental diet was offered to three tanks at 5% of their initial biomass three times daily (08:00 and 12:00, 16:00) for 60 days. 1 h following feeding, uneaten feed was siphoned, dried (70°C), and weighed to determine feed conversion ratio (FCR), protein productive values (PPVs), and lipid productive value (LPVs). The water exchange was approximately 25%, daily. Water temperature, pH, ammonia, and dissolved oxygen were 31.5 ± 1.2°C, 8.2 ± 0.1, < 0.2 ppm, and 5.5 ± 1.0 mg/L, respectively. The photoperiod was artificial, 12 h light: 12 h dark. The SW salinity (35 g/L) was prepared by diluting SW (48 g/L, Khore Musa bay, North-West of Persian Gulf, Khuzestan, Iran) with fresh water in a 10 m^3^ concrete tank. To prepare HS water (50 g/L), sea salt (produced in the same region) was added to the SW in a 10 m^3^ concrete tank. The salinity adjusted water was disinfected and used for the experiment.

### 2.3. Sampling

After 60 days of feeding trial, shrimps were fasted for a day, then shrimps in each tank were first counted and bulk weighed, then their weight and length were individually measured to determine specific growth rate (SGR), weight gain (WG) and Fulton's condition factor (K). During the sampling process, six animals per tank (*n* = 6) were initially kept in chilled (4°C) and aerated SW for 5 min, then they were bled from the ventral sinus using insulin syringes filled with 300 μL anticoagulant (0.01 M trisodium citrate, 0.34 M NaCl, 10 mM EDTA, and 0.12 M glucose). The hemolymph was centrifuged (5000 g × 10 min at 4°C) and stored in a −80°C freezer until the analysis of antioxidant parameters. The HP (*n* = 6 per tank) of the same shrimp was dissected and kept at −80°C to evaluate immune-related genes. For whole-body proximate and FA analysis three shrimp per tank (*n* = 3 per tank) were sampled and stored.

### 2.4. Proximate Composition and FA Profile

Biochemical compositions of the diets and whole body were examined based on standard methods [26]. Se and the other mineral levels, including calcium (ca), potassium (K), magnesium (Mg), phosphorous (P) and sodium (Na) in the whole body were determined by inductively coupled plasma mass spectrometry (ICP-MS). In summary, samples were digested using concentrated nitric acid and hydrogen peroxide, filtered, and diluted with ultrapure water before analysis [28]. FA composition of diets (Table 2) and fish whole body was examined according to Christie [29]. A gas chromatograph (GC) (Agilent Technologies Inc., Santa Clara, CA, USA), equipped with an auto-sampler, a flame ionization detector, and a cyanopropyl-phenyl capillary column (DB-225 MS, 30 m × 0.250 mm inner diameter × 0.25 μm film thickness) was used for FA analysis. An external standard FA methyl ester mixture (GLC-68d; Nu-Chek Prep., Waterville, MN, USA) was used for FA detection.

### 2.5. Antioxidant Enzymes and Lipid Peroxidation

To evaluate antioxidant parameters, shrimp hemolymph was used. Catalase (CAT) [30], and superoxide dismutase (SOD) [31] were measured based on standard protocols. Glutathione (GSH) content was determined following the method of Beutler et al. [32] at 412 nm, expressed in µmol mL/hemolymph. The GPx activity and malondialdehyde (MDA) were carried out using commercial kits (Navand Lab Kit, Navand Salamat Co., Iran).

### 2.6. Hemato-Immunological Parameters

Serum total protein was assessed using a commercial diagnostic kit (Biorex, Shiraz, Iran). Phenoloxidase (PO) activity was determined spectrophotometrically at 490 nm by measuring dopachrome production from L-dihydroxyphenylalanine (L-DOPA), following the method of Hernandez-Lopez et al. [33], using an ELISA reader (BIORAD, USA). At the end of the rearing period, total hemocyte count (THC) and differential hemocyte count were assessed. THC was determined using a Thoma hemocytometer (× 400) [34]. Differential hemocyte counts, including hyaline cells (HCs), semigranular cells (SGCs), and granular cells (GCs), were obtained by: (a) preparing and air-drying hemolymph smears at room temperature (25°C); (b) fixing the smears in methanol for 1 min; (c) staining with the May-Grunwald-Giemsa method; (d) enumerating cell types using a light microscope (Leitz Microscope, Germany).

### 2.7. Gene Expression

Hepatopancreas was homogenized, and total RNA was extracted by an RNA extraction kit (CinnaGen, Tehran, Iran). Then, cDNA was synthesized from 1 µg of the extracted RNA employing a cDNA synthesis kit (CinnaGen, Tehran, Iran) according to the manufacturer's protocol. Quantitative real-time PCR (qRT-PCR) was completed using the SYBR Green Real-time Master Mix (CinnaGen, Tehran, Iran) to calculate the expression of selected biomarkers like insulin-like growth factor hormone (*igf*), lectin (*lec*), peneidine 3a (*pen-3a*), prophenoloxidase (*propo*), and lysozyme (*lyz*) (Table 3). The β-actin gene was used as the internal standard in all tests. The PCR mixtures were run in the Rotor-Gene 3000 cycler (Corbett Life Science, Sydney, Australia) with the following conditions: an initial activating step at 95°C for 1 min, followed by 40 cycles of 15 s at 95°C, 60°C for 1 min, and 72°C for 30 s. The relative expression of the targeted genes was obtained based on the ΔΔCt method described by Schmittgen and Livak [35].

### 2.8. Statistics

All data were presented as means ± standard error. Statistical analysis was carried out using SPSS software (Version 23.0, Chicago, IL, USA). The normality and homogeneity of the variance were assessed by Kolmogorov–Smirnov and Levene tests, respectively. The independent effects of dietary FO, SeNPs, and water salinity and their interactions on growth parameters, body composition, FA profile, antioxidant factors, and immune related genes were determined by a three-way ANOVA. If the effects of independent factors were significant, they were further analyzed individually using one-way ANOVA. Tukey's post test was performed, when significant differences were found among them. The level of significant differences among groups was set at *p* < 0.05 for all statistical tests.

## 3. Results

### 3.1. Growth Performance

Survival rates were over 98% and no significant difference was observed among groups (Table 4, *p* > 0.05). Shrimps in FO_6_Se^0.4^SW and FO_6_Se^0.8^SW exhibited higher growth performance, including final weight, SGR, and WG compared to those in FO_3_Se^0.8^SW and FO_3_Se^0.4^HS, and other experimental treatments showed intermediate values (*p* < 0.05). Final length and somatic factors, including K and hepatosomatic index did not vary among the experimental groups. Feed intake in FO_6_Se^0.4^SW was higher than those in FO_3_Se^0.4^SW and FO_3_Se^0.8^SW groups. FCR, PPV, and LPV did not vary among treatments. Growth performance parameters were significantly affected by FO levels and the interaction among S, FO, and SeNPs levels (*p* < 0.05, Table 5). In addition, FI significantly increased by increasing dietary FO levels (Table 5).

### 3.2. Body Composition

Whole-body moisture in FO_3_Se^0.8^SW was higher than FO_3_Se^0.4^HS and FO_6_Se^0.4^HS (Table 6, *p* < 0.05) and it significantly decreased in HS water (Table 5). Whole-body crude protein in FO_3_Se^0.4^HS, FO_3_Se^0.8^HS, and FO_6_Se^0.4^HS was higher than FO_3_Se^0.8^SW and it significantly increased by HS and FO levels (Table 6). Crude ash in shrimps reared in HS water was higher than SW groups (*p* < 0.05, Table 5). Crude lipid in the whole body did not change among treatments (Table 6). The highest whole- body Se was observed in FO_3_Se^0.8^SW and the lowest values were recorded in FO_6_Se^0.4^SW, FO_3_Se^0.8^HS, and FO_6_Se^0.8^HS (Table 6, *p* < 0.05). These variations were influnced by water salinity, FO level, and their interactive effects (Table 5). The highest Ca levels were found in FO_3_Se^0.4^SW, FO_3_Se^0.8^SW, and FO_6_Se^0.4^SW and the lowest values were in FO_6_Se^0.4^HS and FO_6_Se^0.8^HS (Table 6). These variations were influenced by water salinity, FO levels and their interactions (Table 5). The highest K level was in FO_6_Se^0.8^SW and the lowest values were recorded in FO_6_Se^0.4^HS and FO_6_Se^0.8^HS groups (Table 6, *p* < 0.05). These differences were caused by water salinity and the interaction between water salinity and dietary FO levels. The highest Mg level were in FO_6_Se^0.4^SW and FO_6_Se^0.8^SW groups and the lowest values were in FO_6_Se^0.4^HS and FO_6_Se^0.8^HS groups (Table 6). These variations were influenced by water salinity and the interaction between water salinity and dietary FO levels (Table 5). The highest *p* level was recorded in FO_6_Se^0.8^SW and the lowest values were in FO_6_Se^0.4^HS and FO_6_Se^0.8^HS treatments (Table 6), and these fluctuations were influenced by water salinity, FO level and their interaction (Table 5). Shrimp reared in SW contained higher Na levels compared to those maintained in HS conditions and it was influenced significantly by water salinity (*p* < 0.05).

### 3.3. FA Profile

The highest saturated fatty acid (SFA) levels were observed in FO_3_Se^0.8^SW, FO_3_Se^0.8^HS, and FO_6_Se^0.8^HS, and the lowest level was in FO_3_Se^0.4^SW (*p* < 0.05, Table 7). The SFA levels were affected by SeNPs and the interaction between dietary FO and SeNPs levels (Table 5). The amount of monounsaturated fatty acids (MUFAs) level in FO_3_Se^0.4^SW was higher than FO_3_Se^0.8^SW, FO_6_Se^0.4^SW, FO_3_Se^0.4^HS, and FO_6_Se^0.8^HS. The MUFA levels were influnced by the interaction among dietary FO, SeNPs, and water salinity (Table 5). Shrimp from the FO_3_Se^0.4^SW and FO_6_Se^0.4^SW groups showed higher levels of ALA, 18:2n − 6 and n − 6 PUFAs compared to the FO_3_Se^0.8^SW, FO_3_Se^0.8^HS, and FO_6_Se^0.8^HS groups. Furthermore, the 0.4 mg/kg SeNPs supplementation level enhanced the concentration of these FAs. The highest level of ARA was observed in FO_6_Se^0.8^SW, and the lowest levels in FO_3_Se^0.8^SW and FO_3_Se^0.8^HS. ARA was affected by dietary FO levels and interaction between FO and SeNPs. In contrast, LA (18:3n − 3) was highest in FO_6_Se^0.8^HS, and its amount increased by 0.8 mg/kg SeNPs supplementation levels. The amount of n − 3 PUFA was highest in FO_6_Se^0.4^SW and FO_3_Se^0.4^HS and it was affected by water salinity, SeNPs levels and interaction among dietary FO, water salinity, and SeNPs levels. The level of EPA was highest in FO_3_Se^0.4^SW, FO_6_Se^0.4^SW, and FO_3_Se^0.4^HS, and it increased by 0.4% SeNPs supplementation levels (Table 5). The amount of DHA and LC-PUFA in FO_3_Se^0.4^SW, FO_6_Se^0.4^SW, FO_6_Se^0.8^SW, and FO_6_Se^0.4^HS were relatively higher than the other treatments. DHA and LC-PUFA levels decreased with both increasing water salinity and dietary supplementation of 0.8 mg/kg SeNPs. In contrast, 6% FO supplementation enhanced their accumulation.

### 3.4. Antioxidant Capacity

**Figure 1:**
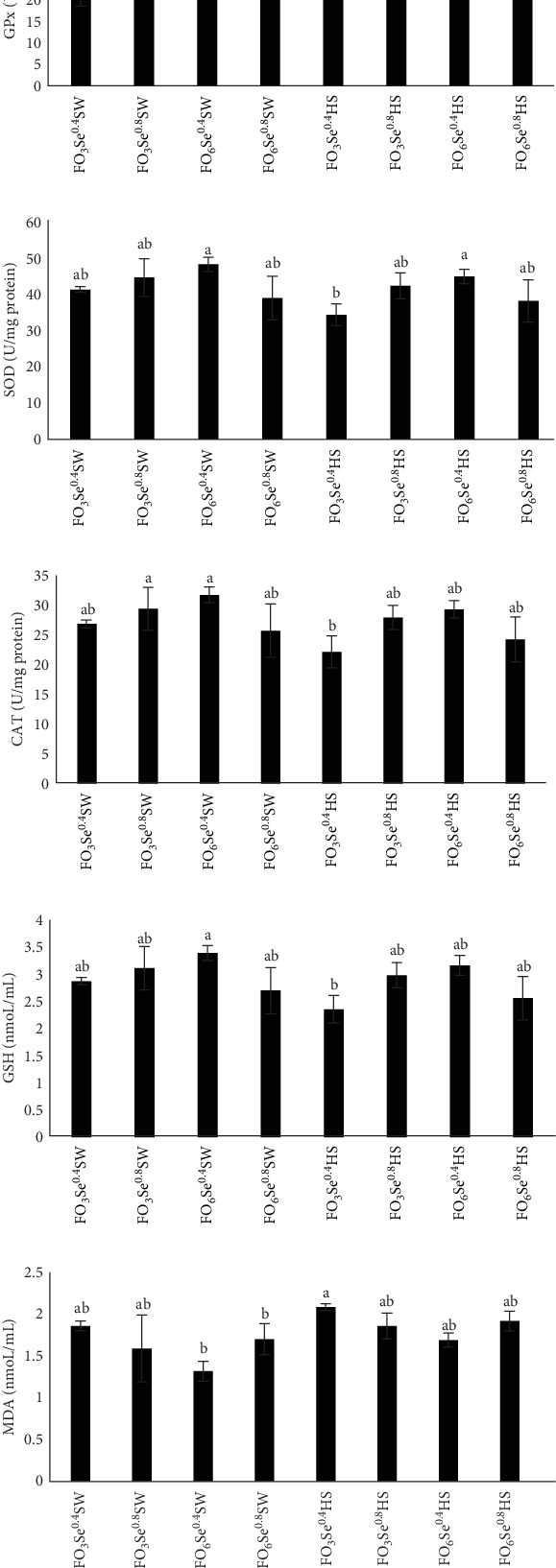
Antioxidant status of the hemolymph in *P. vannamei* fed different diets over 60 days, including glutathione peroxidase activity (A), superoxide dismutase activity (B), catalase activity (C), glutathione content (D), and malondialdehyde level (E). Values are presented as mean ± SE, *n* = 3. Values with different letters are significantly different (*p* < 0.05) based on Tukey's HSD test following three-way ANOVA.

The highest and lowest levels of GPx (Figure 1A) activities were in FO_6_Se^0.4^SW and FO_3_Se^0.4^HS, respectively, and it decreased by increasing water salinity and dietary 3% FO level and was also affected by the interaction between dietary FO and Se levels (p < 0.05). The highest SOD value was observed in the FO_6_Se^0.4^SW and FO_6_Se^0.4^HS groups, while the lowest levels occurred in FO_3_Se^0.4^HS (Figure 1B). SOD activity decreased with increasing water salinity and was modulated by the interactive effects of FO and SeNP levels. The highest values of CAT (Figure 1C) were observed in FO_6_Se^0.4^SW and FO_6_Se^0.8^Ws, and the lowest level was in FO_3_Se^0.4^HS and it was suppressed by increasing water salinity and was also affected by the interaction between dietary FO and Se levels. The highest and lowest levels of GSH (Figure 1D) were in FO_6_Se^0.4^SW and FO_3_Se^0.4^HS, respectively, and were affected by interaction between water salinity and dietary FO or Se levels (p < 0.05). The lowest MDA levels were observed in the FO_6_Se^0.4^SW and FO_6_Se^0.8^SW groups, while the highest concentrations occurred in FO_3_Se^0.4^HS (Figure 1E). MDA levels increased with elevated water salinity and were modulated by the interactive effects of FO and SeNP supplementation.

### 3.5. Hemato-Immunological Parameters

Serum immune factors, including total protein and PO activity did not varey significantly among treatments (Table 8, *p* > 0.05). In addition, total hemocytes count and its differentiation were not affected by any of the experimental treatments.

### 3.6. Gene Expression

**Figure 2:**
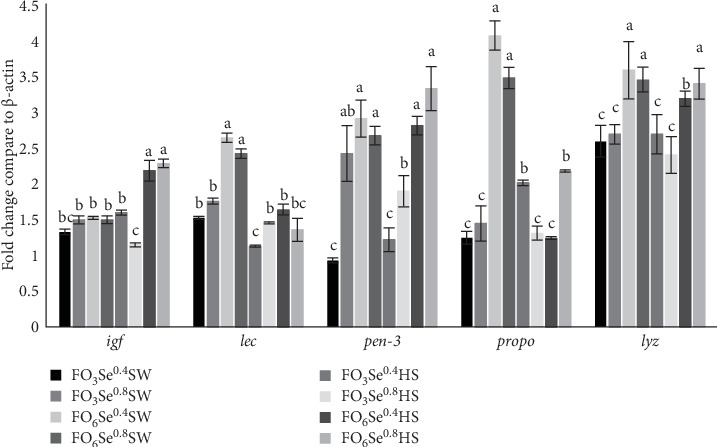
The expression level of different genes, including insulin-like growth hormone, lectin, peneidine 3, prophenoloxidase, and lysozyme in hepatopancreas of *P. vannamei* fed the experimental diets over 60 days. Values are presented as mean ± SE, *n* = 3. Values with different letters are significantly different (*p* < 0.05) based on Tukey's HSD test following three-way ANOVA.

The amount of *igf-1* (Figure 2) gene transcription level in FO_6_Se^0.4^HS and FO_6_Se^0.8^HS was higher than the other groups (p < 0.05) and it increased by water salinity and dietary FO level and their interaction (Table 5). The FO_6_Se^0.4^SW and FO_6_Se^0.8^SW groups exhibited the highest *lec* transcription levels, while FO_3_Se^0.4^HS showed the lowest expression (Figure 2). The *lec* expression was suppressed by both increasing water salinity and decreasing dietary FO levels. The level of *pen-3a* gene transcription level increased in FO_6_Se^0.4^SW, FO_6_Se^0.8^SW, FO_6_Se^0.4^HS, and FO_6_Se^0.8^HS, and it was enhanced by increasing dietary FO and Se levels. The highest *propo* gene transcription levels were observed in the FO_6_Se^0.4^SW and FO_6_Se^0.8^SW groups. The *propo* expression increased with higher FO levels and was modulated by interactive effects of water salinity, dietary FO, and SeNP supplementation. (Table 5). The highest amounts of *lyz* gene transcription levels were in FO_6_Se^0.4^SW, FO_6_Se^0.8^SW, and FO_6_Se^0.8^HS and were affected by water salinity, dietary FO, and Se levels and their interaction (Figure 2, *p* < 0.05).

## 4. Discussion

### 4.1. Growth Performance

While *P. vannamei* is a euryhaline crustacean and can resist high salinity fluctuations, extra deviation of osmotic pressure of rearing water from the isotonic point may adversely affects their survival and physiological performance and leads to increased mortality and growth retardation [18, 36]. *P. vannamei* has limited capacity to synthesize LC-PUFA and convert PUFA precursors to LC-PUFA [20, 37, 38], thus, dietary FO has superior nutritional value as it provides both n − 3 and n − 6 LC-PUFA [39, 40]. In this context, Hurtado et al. [41] reported that LC-PUFA deficient diet reduced *P. vannamei* performance reared at 50 g/L; however, supplementing diet with high LC-PUFA levels improved growth at high salinities (30–50 g/L). In addition, low salinity (3 g/L) negatively affected *P. vannamei* survival and growth, especially on diets with LC-PUFA deficiency; however, dietary FO improved survival under osmotic stress by promoting osmoregulation, Na^+^/K^+^-ATPase and total ATPase activities and modulating water and ion permeability [22].

The optimal dietary Se level for *P. vannamei* growth varies depending on various parameters, including the source/form ingested, the time of administration, the experimental condition, and the levels of LC-PUFA or the presence of other antioxidants (i.e., vitamin E) in the aquafeed [14, 17, 42]. In the current study, the interactive effects of dietary SeNPs, FO, and water salinity levels affected *P. vannamei* growth performance indicating an interconnection among these parameters. The combination of high levels of FO and SeNPs enhanced the growth performance of *P. vannamei* in HS conditions. In contrast, antagonistic effects were observed between low dietary FO and high SeNPs levels in SW, as well as between low FO and SeNPs levels in HS. Specifically, growth performance in the FO_3_Se^0.4^HS group, reared in HS water, declined potentially due to osmotic stress in this group. However, increasing dietary SeNPs and FO levels improved growth under HS conditions. On other hand, growth rate of *P. vannamei* in FO_3_Se^0.8^SW decreased, which could be associated with toxic effects of high SeNPs at low LC-PUFA levels in *P. vannamei* reared in SW (35 g/L). In this context, Ghaffarizadeh et al. [17] reported that dietary extra SeNPs (1.2 mg/kg) suppressed growth and antioxidant enzymes activity and resulted in oxidative stress by increasing lipid peroxidation level in HP. Furthermore, Yu et al. [16] found that a diet containing 0.81 mg/kg of inorganic se (Na selenite) induced toxic effects in *P. vannamei*, leading to endoplasmic reticulum stress and damage to the HP. High dietary intake of Se can lead to toxicity through the overproduction of seleno-sulfur amino acids, such as selenocysteine and selenomethionine, which may impair the function of Se-dependent antioxidant enzymes [7]. Interestingly, shrimps fed the same diet had better growth performance in HS (50 g/L) conditions, indicating higher dietary Se is essential in HS water. It has been confirmed that dietary Se may improve growth by enhancing growth hormone, thyroxine production, antioxidant capacity, cell membrane protection, protein digestibility, and stimulation of protein synthesis in intestinal cells [18, 43]. In this context, Yu et al. [16] reported that L-selenomethionine and se yeast (0.4 mg/kg) improved growth compared to Na selenite and SeNPs. In another study, Yu et al. [14] reported that under low salinity stress (3 g/L), survival rate, WG, and HP index increased in *P. vannamei* fed 0.84 mg/kg L-selenomethionine, suggesting higher Se requirements for this species under osmotic stress. Also, Karamzadeh et al. [44] reported that *P. vannamei* fed diets supplemented with SeNPs (0.3 mg/Kg) or zinc nanoparticles (0.3 mg/Kg) or their combination (0.15 SeNPs + 0.15 ZnNPs) showed improved growth, survival, antioxidant activity, and immune response compared to control groups. More studies at molecular level are required to elucidate the mode of action and the interaction among dietary Se, LC-PUFA, and rearing conditions in *P. vannamei*.

### 4.2. Biochemical Composition

The results of the present study showed that rearing shrimp in HS water increased whole-body protein and ash content while reducing moisture levels. This reduction in moisture percentage appears to have resulted in the increment of crude protein and ash content. In line with our results, Huang et al. [45] demonstrated that as ambient salinity increased, the water content of *P. vannamei* decreased, while lipid and/or protein contents increased. In contrast, Li et al. [46] reported that increasing water salinity from 3 to 32 g/L increased whole-body moisture and reduced protein and lipid levels. In addition, Ghaffarizadeh et al. [17] reported that while dietary SeNPs significantly decreased whole-body lipid content in *P. vannamei*, it did not affect lipid levels in this species. Yu et al. [14] reported that whole-body ash decreased in *P. vannamei* juveniles fed 0.84 mg/Kg reared in 3 g/L water compared to those reared in 31 g/L water; however, other biochemical parameters did not change. Also, Said et al. [47] reported that supplemented diet with SeNPs extracted from *Spirulina platensis* increased whole-body protein and ash but reduced lipid content.

In the present study, whole-body Se content mainly reduced in shrimp reared in HS water suggesting its higher metabolism to synthetize GPx during osmotic stress. In contrast, in this study the combination of high dietary SeNPs level and rearing in SW increased whole-body Se retention in shrimp. In addition, whole-body Se decreased with increasing dietary FO level indicating its consumption to protect cell membranes in the presence of high amount of LC-PUFA. In contrast, previous studies reported that whole-body Se increased with increasing dietary Se in juvenile oriental river prawn [9], smooth marron (*Cherax cainii*) [13], and *P. vannamei* [17]. The results of present study showed that rearing shrimp in HS water pronouncedly reduced whole-body macro elements content (i.e., Ca, K, Mg, P, and Na), suggesting ionic homeostasis malfunction during rearing in HS water. In this context, it has been suggested that *P. vannamei* excretes body Ca in HS conditions to maintain homeostasis [48]. Stevenson [48] stated that the crustaceans reared in freshwater may store Ca or other macroelements in tissues for cuticular mineralization. However, in the saline waters with higher content of Ca, they may not require to store Ca in the body. In this context, Jannathulla et al. [23] reported that increasing water salinity from 3 to 60 g/L reduced whole-body moisture, ash, Ca, Mg, P, and K, but increased whole-body protein and lipid levels in *P. vannamei*. The authors also reported that whole-body Na decreased by increasing salinity from 3 to 40 g/L, then increased from 50 to 60 g/L. Interestingly, in the present study the synergistic effects of high dietary SeNPs and rearing in SW conditions increased Ca and P contents in the shrimp whole-body. In addition, the combination of high dietary FO and rearing in SW elevated K and Mg levels, meanwhile increasing dietary FO at HS water, pronouncedly decreased their levels. These results exhibits dual role of LC-PUFA in shrimp osmoregulation, based on the level of water salinity. These results show that whole-body proximate composition is not only affected by feed ingredients, but also significantly affected by ambient factors such as water salinity.

### 4.3. FA Profile

The results of the present study showed that whole-body FA profile was not only affected by dietary FO level, but also was markedly influenced by dietary SeNPs and water salinity levels. For instance, the percentage of SFA in the whole body increased with increasing dietary SeNPs to 0.8 mg/kg suggesting high amount of SeNPs may have negative effects on FA profile of shrimp. In our study, the amount of ALA increased in shrimp-fed diets containing 0.4 mg/kg SeNPs. Additionally, increasing dietary FO to 6% enhanced whole-body ARA and DHA, as FO serves as the main source of these FA. Previous studies on *P. vannamei* showed that shrimp-fed FO-based diets contained higher DHA and EPA in their tissues [20, 37, 38]. Furthermore, supplementing diet with 0.4 mg/kg SeNPs also increased ARA retention. Also, the amount of EPA, DHA, n − 3 PUFA, and LC-PUFA increased by 0.4 mg/kg SeNPs level, indicating supplementing diet with 0.4 mg/kg SeNPs had positive effects on FA profile of shrimp. Furthermore, there was an antagonistic effectbetween low dietary FO (3%) and high SeNPs (0.8 mg/kg) levels that reduced EPA, DHA, and LC-PUFA in shrimp whole body. In this regard, Tseng et al. [25] showed that supplementing diet with hydroxy-selenomethionine (0.79 mg Se kg diet^−1^) increased n − 3 LC-PUFA and DHA retention in gilthead sea bream. Moreover, Jafari et al. [49] observed that the amount of DHA in the whole body of Arabian yellowfin seabream (*Acanthopagrus arabicus*) larvae increased by increasing SeNPs level in diet. However, in the present study, the extra level of SeNPs increased SFA and decreased LC-PUFA retention in shrimp whole body, suggesting β-oxidation of LC-PUFA increased by supplementing diet with 0.8 mg/kg SeNPs. Moreover, in the current study the amount of DHA, n − 3 PUFA, and LC-PUFA decreased by rearing shrimp in HS water, indicating these FAs may be metabolized to keep shrimp homeostasis and gill membrane fluidity in HS water. These variations in FA profile may be linked to osmoregulation mechanisms involving biomembrane structure, mitochondrial function, energy supply, and hemolymph osmolytes [48]. In this regard, Palacios et al. [21] reported that the amount of LC-PUFA in gills of *P. vannamei* increased with reducing water salinity from 35 to 10 g/L. A possible explanation could be related to the selective oxidation rate of FA and also the FA transfer between polar and neutral lipids [21]. In contrast, Hurtado et al. [41] reported that in HP of *P. vannamei*-fed low LC-PUFA content diet, increasing water salinity from 30 to 50 g/L, LC-PUFA particularly ARA, EPA, and DHA reduced in neutral fraction of lipids. However, the authors reported that the amount of LC-PUFA did not change when shrimp was fed high LC-PUFA content diets. In contrast, Chen et al. [22] reported that the amount of n − 3 PUFA, EPA, and DHA increased with increasing water salinity from 3 to 30 g/L. Also, Ye et al. [50] reported that increasing water salinity from 1 to 15 g/L increased whole-body LC-PUFA level in low-salinity-tolerant hybrid and normal variety of *P. vannamei*. Further research is required to evaluate the FA profile in different tissues, particularly the gills, by analyzing both neutral and polar lipids. This will help to assess the potential transfer of FAs between them, when shrimps are reared at varying water salinities.

### 4.4. Antioxidant Enzymes

Irrespective of the dietary Se form, it metabolizes into selenocysteine, which is then used to synthetize selenoproteins such as GPx with antioxidative properties [6]. Among various Se sources, SeNPs showed superior antioxidant capabilities due to its higher efficiency [51]. This is because SeNPs boosts the production of GPx and activates a chain reaction of antioxidant enzymes [51]. In the present investigation, rearing shrimp in HS water pronouncedly suppressed antioxidant enzymes activities and increased MDA values, especially in FO_3_Se^0.4^HS group, suggesting HS water (50 g/L) may induce oxidative stress in *P. vannamei*. In this context, it has been suggested that acute salinity stress could increase ROS concentration and result in oxidative stress [52]. In addition, in our research the combination of low dietary SeNPs and high FO levels (FO_6_Se^0.4^) improved antioxidant enzymes activities and reduced MDA level in both SW and HS conditions. Also interestingly, increasing dietary FO reduced MDA level, particularly in FO_6_Se^0.4^SW and FO_6_Se^0.8^SW groups, suggesting appropriate LC-PUFA levels could result in better antioxidant capacity in shrimp. In this context, elevated SOD activity was also observed in *P. vannamei*-fed diets supplemented with FO [53]. In contrary, an antagonistic effect between dietary low SeNPs and low FO levels in FO_3_Se^0.4^HS not only reduced growth but also suppressed antioxidant enzymes activity and increased whole-body MDA in this group. Previous studies in *P. vannamei* have shown that dietary SeNPs, resulted in improved antioxidant capacity and reduced MDA level in this species [16, 17]. In addition, Yu et al. [14] reported that diets with 0.84 and 1.14 mg/kg of L-selenomethionine provided strong antioxidant activity and enhanced stress tolerance to osmotic challenge test in *P. vannamei*. Furthermore, Karamzadeh et al. [44] reported that shrimp-fed SeNPs or SeNPs + Zn-NPs revealed higher CAT, SOD, and GPx activities in *P. vannamei*. Also, Said et al. [47] reported that dietary SeNPs derived from *Spirulina platensis* improved the antioxidant capacity in both muscles and HP of *P. vannamei*, evidenced by reduced MDA level and elevated CAT, SOD, TAC activity. Finally, Mohtashemipour et al. [54] reported that supplementing diet with 2–4 mg SeNPs/kg diet significantly increased antioxidant capacity in Asian seabass (*Lates calcarifer*) reared in HS water. Further studies are required to assess the mode of action of dietary LC-PUFA and SeNPs at molecular level.

### 4.5. Gene Expression

Insulin growth factor-1 is an essential regulator of development, growth, metabolism, as well as stress resistance in many organisms. The involvement of IGF signaling in salinity stress was also reported in crustaceans such as Chinese mitten crab (*Eriocheir sinensis*) [55]. In the present study, the combination of dietary high FO and rearing in HS, triggered *igf* gene transcription in FO_6_Se^0.4^HS and FO_6_Se^0.8^HS, suggesting shrimps in these groups exhibited better adaptation to HS water. In addition, rearing shrimp in HS water induced *igf* in *P. vannamei*, suggesting an integral role of this hormone in osmoregulation. Furthermore, increasing dietary FO resulted in higher *igf* gene transcription level, suggesting dietary LC-PUFA may exert its effects on osmoregulation through modulation of *igf* gene expression.

lecs are a class of pattern recognition proteins and they can identify and bind to glycan structures present on the surface of pathogens and interact with pathogen-associated molecular patterns to initiate a range of immune responses, such as hemocyte recruitment, opsonization, and immune-related genes regulation [56]. In the present study, *lec* gene transcription levels increased in FO_6_Se^0.4^SW and FO_6_Se^0.8^SW, which correlated with improved growth performance in these groups. Conversely, rearing shrimps in HS water down-regulated *lec* gene expression, suggesting that osmotic stress may suppress certain immune responses.

Penaeidins are antimicrobial peptides that are produced and stored in granular hemocytes, playing an integral role in crustacean defense [57]. In the present study, irrespective of water salinity, shrimps-fed FO_6_Se^0.4^ and FO_6_Se^0.8^ diets exhibited higher *pen-3* gene transcription levels than the other groups, indicating high LC-PUFA levels can trigger immune-related gene expression in *P. vannamei*.

The propo-activating cascade acts as a nonself-recognition system that identifies pathogen-associated molecular patterns on bacterial and fungal cell walls. This recognition attracts hemocyte and induces melanization to inactivate pathogenic cells [58]. In the present study, *propo* transcription level increased in FO_6_Se^0.4^SW and FO_6_Se^0.8^SW, which was in concomitant with up-regulation of *lec*, *pen-3*, and *lyz* suggesting interactive effects of dietary FO and SeNPs on immune responses of shrimp. However, PO activity, total hemocytes count or its differentiation did not change among treatments, indicating post transcriptional regulation. In contrast, Ghaffarizadeh et al. [17] reported that dietary supplementation with 0.2–0.4 mg/kg SeNPs enhaned PO activity, which was associated with up-regulation of *propo* gene expression in *P. vannamei*. Furthermore, Mohtashemipor et al. [54] reported that supplementing diet with 2–4 mg/kg SeNPs triggered nonspecific hummoral immune responses in *L. calcarifer* reared in HS water. These discrepancies among studies may be attributed to variations in dietary composition, husbandry conditions, and water salinity levels.

*lyz*, a nonspecific immune factor, targets the peptidoglycan layer of Gram-positive bacteria and as an opsonin, promotes phagocytosis of Gram-negative bacteria [59]. The results of our study showed elevated transcription levels increased in the FO_6_Se^0.4^SW, FO_6_Se^0.8^SW, and FO_3_Se^0.8^HS, which were linked to the individual and interactive effects of dietary FO, SeNPs, and water salinity. Specifically, higher dietary FO and SeNP levels induced *lyz* gene expression, whereas HS conditions down-regulated it, indicating immunosuppressing effects of osmotic stress. In this regard, Ghaffarizadeh et al. [17] demostrated that dietary SeNPs up-regulated the mRNA transcript abundance of *propo*, *lyz*, and *pen-3a in P. vannamei*, indicating that se modulates and enhances immune responses. Similarly, Yang et al. [60] reported increased *lyz* expression in in Chinese mitten crabs fed 1.5 mg/kg organic Se, whereas higher doses (3.0 mg/kg Se) down-regulated its expression. Further proteomix studies are required to evaluate the interactive influence of dietary SeNPs and LC-PUFA levels under osmotic sress, to shed light on their mode of action.

## 5. Conclusion

In summary, the findings of the current investigation showed that the interactive effects of dietary SeNPs and FO can improve growth, antioxidant capacity, immune responses, and stress resistance of *P. vannamei* reared in HS water conditions. The results of the present study showed that increasing dietary FO levels from 3% to 6% improved the growth performance of shrimps reared in both SW and HS conditions. Additionally HS water significantly increased whole-body protein and ash contents while reducing moisture levels. Furthermore, supplementation with 0.4 mg/kg SeNPs enhanced whole-body acid profile, as evidenced by increased levels of EPA, DHA, n − 3 PUFA and LC-PUFA, indicating an appropriate SeNP dose for improving shrimp FA profile. Shrimps reared in HS water exhibited suppressed antioxidant enzymes activity accompanied by elevated MDA levels, indicating oxidative stress. However, supplementing diet with 6% FO and SeNPs, alleviated these negative effects. Finally, the transcription levels of *igf* and immune-related genes increased by increasing dietary FO level to 6% and also modulated by the interaction of water salinity, dietary SeNPs, and FO levels. Based on the findings of the current study, the antagonistic effects between low dietary FO (3%) and high SeNPs (0.8 mg/kg) in SW or dietary low FO (3%) and low SeNPs (0.4 mg/kg) in HS, resulted in reduced growth performance, and suppressed antioxidant capacity in the experimental shrimps. Meanwhile, the combination of high dietary FO (6%) and low level of SeNPs (0.4 mg/kg), was found to enhance *P. vannamei* tolerance to rearing in HS water (Figure S1).

## Figures and Tables

**Table 1:** Experimental feeds formulation (g/kg) and proximate composition (%).

Ingredients^a^	Experimental diets			
	FO_3_Se^+0.4^	FO_3_Se^+0.8^	FO_6_Se^+0.4^	FO_6_Se^+0.8^
Fish meal	200	200	200	200
Squid meal	35	35	35	35
Spirulina meal	15	15	15	15
Shrimp meal	50	50	50	50
Soybean meal	150	150	150	150
Corn gluten	50	50	50	50
Wheat gluten	50	50	50	50
Soybean isolate	50	50	50	50
Wheat middling	230	230	230	230
Beef gelatin	40	40	40	40
DL-methionine	5	5	5	5
L-lysine	10	10	10	10
Fish oil	30	30	60	60
Soybean oil	30	30	—	—
Soy lecithin	20	20	20	20
Yeast	10	10	10	10
Vitamin premix^b^	10	10	10	10
Mineral premix^c^	10	10	10	10
Dicalcium phosphate	5	5	5	5
Selenium nanoparticles (mg)	0.4	0.8	0.4	0.8
Proximate composition				
Protein	43.2	43.0	42.8	43.1
Lipid	11.2	11.6	11.6	11.2
Ash	10.5	10.3	10.5	10.6
Moisture	8.0	9.0	8.5	8.5
LC-PUFA	0.6	0.7	1.2	1.2
Selenium (mg/kg)	0.8	1.3	0.9	1.2

^a^All these ingredients were supplied by Taam Sazan Co (Bushehr, Iran).

^b^Vitamin premix (IU/kg of premix): ascorbic acid, 350,000; retinol, 1,000,000,000; cholecalciferol, 500,000,000; tocopherols, 500,000; vitamin K3, 960,000; vitamin B1, 980,000; vitamin B2, 800,000; vitamin B6, 990,000; vitamin B9, 950,000; vitamin B12, 10,000; vitamin B7, 20,000; vitamin B3, 995,000; vitamin B5, 980,000.

^c^Mineral premix (IU/kg of premix): MnSO_4_, 330,000; MnO, 240,000; Fe_2_(SO_4_)^3^, 210,000; ZnO, 800,000; CuSO_4_, 240,000; Ca(IO_3_)^2^ H_2_O, 620,000; MgSO_4_, 10,000; CrCl_3_, 990,000; KCl, 880,000, choline chloride, 600,000; inositol, 980,000; CaCO_3_, 990,000.

**Table 2:** The fatty acid profile (% of extracted lipids) of the experimental feeds.

Ingredients	Experimental diets			
	FO_3_Se^+0.4^	FO_3_Se^+0.8^	FO_6_Se^+0.4^	FO_6_Se^+0.8^
16:0	15.7	15.4	19.6	18.8
18:0	4.3	4.3	5.2	5.1
SFA	22.4	22.1	27.9	26.8
18:1n − 9	41.6	41.6	32.5	32.0
MUFA	44.6	44.7	37.2	36.5
18:2n − 6	24.3	23.9	22.8	24.3
18:3n − 3	3.1	3.3	1.8	1.7
20:4n − 6	0.4	0.4	0.3	0.3
20:5n − 3	1.3	1.3	2.7	2.7
22:6n − 3	2.6	2.7	5.5	5.4
n − 6 PUFA	25.3	24.9	23.9	25.6
n − 3 PUFA	7.2	7.5	10.2	10.2
LC-PUFA	5.4	5.7	10.1	10.1
n − 3/n − 6	0.3	0.3	0.4	0.4
DHA/EPA	2.0	2.0	2.0	2.1
ARA/EPA	0.3	0.3	0.1	0.1

*Note:* SFA, saturated fatty acids includes: 14:0, 15:0, 17:0, and 20:0. MUFA, monounsaturated fatty acids includes: 14n − 1, 16n − 7, 18n − 7, and 20:1n − 4. PUFA, polyunsaturated fatty acids includes: 20:3n − 6, 22:4n − 6, 22:5n − 6, and 22:5n − 3. LC-PUFA, long-chain polyunsaturated fatty acids includes: 20:3n − 6, 20:4n − 6, 20:5n − 3, 22:4n − 6, 22:5n − 6, 22:5n − 3, and 22:6n − 3.

**Table 3:** Primer sequences used for gene expression analysis in *P. vannamei* juveniles using qRT-PCR.

Gene	Accession number	Primers (forward/reverse) sequence	Amplicon bp	Efficiency (%)
Prophenoloxidase	AY723296	F: CGGTGACAAAGTTCCTCTTC R: GCAGGTCGCCGTAGTAAG	122	97
Lysozyme	AY170126.2	F: TGT TCC GAT CTG ATG TCC R: GCT GTT GTA AGC CAC CC	121	98
Insulin-like growth factor	XM02739466	F: CTCTGTACAGTCAGCCCAGC R: CACACCCAGTCAGTCCCAAG	220	98
Lectin	EF583939.1	F: TGA CCC GTG CAA GAC CCA CA R: CCT GTG GAA CTG CCC TGT TGT TT	182	94
Penaeidin-3a	Y14926	F: CACCCTTCGTGAGACCTTTG R: AATATCCCTTTCCCACGTGAC	123	96
Beta actin	AF300705.2	F: CCACGAGACCACCTACAAC R: AGCGAGGGCAGTGATTTC	142	97

**Table 4:** Growth performance of *P. vannamei* juveniles fed various levels of FO and SeNP at various salinity for 56 days.

Parameters	Experimental groups^1^								
	FO_3_Se^0.4^SW	FO_3_Se^0.8^SW	FO_6_Se^0.4^SW	FO_6_Se^0.8^SW	FO_3_Se^0.4^HS		FO_3_Se^0.8^HS	FO_6_Se^0.4^HS	FO_6_Se^0.8^HS
IBW (g)	2.4 ± 0.0	2.4 ± 0.0	2.4 ± 0.0	2.4 ± 0.0	2.4 ± 0.0	2.4 ± 0.0		2.4 ± 0.0	2.4 ± 0.0
IBL (cm)	7.1 ± 0.0	7.1 ± 0.0	7.1 ± 0.0	7.1 ± 0.0	7.1 ± 0.0	7.1 ± 0.0		7.1 ± 0.0	7.1 ± 0.0
FBW (g)	12.1 ± 0.1^ab^	11.6 ± 0.2^b^	12.2 ± 0.1^a^	12.3 ± 0.1^a^	11.5 ± 0.2^b^	12.0 ± 0.2^ab^		12.1 ± 0.2^ab^	11.9 ± 0.1^ab^
FBL (cm)	12.3 ± 0.1	12.1 ± 0.1	12.4 ± 0.1	12.2 ± 0.1	12.2 ± 0.1	12.1 ± 0.1		12.4 ± 0.1	12.2 ± 0.1
SGR (%/day)^2^	2.89 ± 0.0^ab^	2.82 ± 0.1^b^	2.90 ± 0.0^a^	2.92 ± 0.0^a^	2.80 ± 0.0^b^	2.87 ± 0.0^ab^		2.89 ± 0.1^ab^	2.86 ± 0.0^ab^
WG (%)^3^	404.2 ± 4.2^ab^	384.7 ± 8.6^b^	409.7 ± 5.5^a^	413.9 ± 8.5^a^	381.9 ± 8.4^b^	398.6 ± 3.8^ab^		404.6 ± 8.7^ab^	396 ± 4.4^ab^
HPI (%)^4^	4.5 ± 0.2	4.7 ± 0.1	4.4 ± 0.1	4.5 ± 0.2	4.3 ± 0.2	4.6 ± 0.1		4.2 ± 0.2	4.4 ± 0.1
K (%)^5^	0.65 ± 0.0	0.62 ± 0.0	0.64 ± 0.0	0.64 ± 0.0	0.63 ± 0.0	0.63 ± 0.0		0.60 ± 0.0	0.64 ± 0.0
FI (g/fish)^6^	13.8 ± 0.2^b^	13.7 ± 0.0^b^	15.2 ± 0.6^a^	14.6 ± 0.8^ab^	14.5 ± 0.2^ab^	14.3 ± 0.1^ab^		14.6 ± 0.3^ab^	14.7 ± 0.3^ab^
FCR^7^	1.1 ± 0.0	1.2 ± 0.0	1.2 ± 0.1	1.2 ± 0.0	1.3 ± 0.0	1.2 ± 0.0		1.2 ± 0.1	1.2 ± 0.0
PPV^8^ (%)	35.6 ± 3.7	37.3 ± 0.7	35.5 ± 0.5	33.7 ± 2.5	35.8 ± 3.3	32.5 ± 2.0		38.4 ± 2.1	38.0 ± 3.8
LPV^9^ (%)	3.6 ± 0.6	3.9 ± 0.2	3.2 ± 0.2	3.0 ± 0.5	3.7 ± 0.7	3.4 ± 0.2		3.5 ± 0.4	3.4 ± 0.4
SUR^10^ (%)	98.3 ± 1.7	100 ± 0.0	100 ± 0.0	100 ± 0.0	100 ± 0.0	100 ± 0.0		98.3 ± 1.7	98.3 ± 1.7

*Note:* Data are presented as the mean ± pooled SE of three replicates. Values within a column with a common superscript letter are not significantly different from the other dietary groups (*p* > 0.05). The significance of the two main effects (fish meal replacement level and acidifier level) and their interaction were analyzed using two-way ANOVA.

Abbreviations: FBL, final body length; FBW, final body weight; FCR, feed conversion ratio; FI, feed intake; HPI, hepatosomatic index; IBL, initial body length; IBW, initial body weight; LPV, lipid productive value; PPV, protein productive value; SGR, specific growth rate; SUR, survival; WG, weight gain.

^1^Experimental groups: FO_3_Se^0.4^SW (3%FO + 0.4 mg/kg SeNPs reared in SW), FO_3_Se^0.8^SW (3%FO + 0.8 mg/kg SeNPs reared in SW), FO_6_Se^0.4^SW (6%FO + 0.4 mg/kg SeNPs reared in SW), FO_6_Se^0.8^SW (6%FO + 0.8 mg/kg SeNPs reared in SW), FO_3_Se^0.4^HS (3%FO + 0.4 mg/kg SeNPs reared in HS), FO_3_Se^0.8^HS (3%FO + 0.8 mg/kg SeNPs reared in HS), FO_6_Se^0.4^HS (6%FO + 0.4 mg/kg SeNPs reared in HS), and FO_6_Se^0.8^HS (6%FO + 0.8 mg/kg SeNPs reared in HS).

^2^SGR = ([Ln (FBW) − Ln (IBW)]/feeding period (42 days)) × 100.

^3^WG = [FBW (g) − IBW (g)/IBW (g)] × 100.

^4^HPI = Liver weight (g)/body weight (g) × 100.

^5^K = Fulton's condition factor = FBW (g)/L 3 × 100

^6^FI = Total feed intake (g)/number of fish.

^7^FCR = FI (g)/WG (g) × 100.

^8^PPV = Protein gained (g)/protein intake (g) × 100.

^9^LPV = Lipid gained (g)/lipid intake (g) × 100.

^10^SUR = Final number of fish/initial number of fish × 100.

**Table 5:** The individual and interactive effects of FO, SeNP, and water salinity on various physiological responses of *P. vannamei* juveniles.

Parameters	Experimental groups^a^						
	S	FOL	SeL	S × FOL	S × SeL	FO × SeL	S × FOL × SeL
IBW	1.000	1.000	1.000	1.000	1.000	1.000	1.000
IBL	1.000	1.000	1.000	1.000	1.000	1.000	1.000
FBW	0.174	**0.017**	0.689	0.455	0.257	0.990	**0.030**
FBL	0.081	0.374	0.721	0.545	0.183	0.349	0.534
SGR	0.336	**0.006**	0.827	0.646	0.443	0.973	**0.041**
WG	0.078	**0.005**	0.672	0.390	0.180	0.923	**0.010**
HPI	0.423	0.360	0.161	0.752	0.224	0.938	0.684
K	0.071	0.900	0.949	0.541	0.076	0.082	0.455
FI	0.526	**0.025**	0.508	0.18	0.541	0.508	0.508
FCR	0.182	0.338	0.608	0.332	0.978	0.985	0.101
PPV	0.712	0.557	0.612	0.130	0.629	0.940	0.387
LPV	0.863	0.277	0.795	0.427	0.674	0.775	0.642
SUR	0.572	0.572	0.572	0.102	0.443	0.572	0.572
Moisture	**0.001**	0.680	0.169	0.155	0.377	0.625	0.641
Crude protein	**0.001**	**0.034**	0.191	0.117	0.389	0.506	0.358
Crude lipid	0.265	0.816	0.860	0.465	0.741	0.775	0.623
Crude ash	**0.001**	0.071	0.156	0.674	0.454	0.906	0.150
Se	**0.001**	**0.018**	0.678	0.448	**0.001**	0.367	0.578
Ca	**0.001**	**0.002**	0.031	0.341	**0.008**	0.158	**0.014**
K	**0.001**	0.712	0.791	**0.006**	0.146	0.665	0.210
Mg	**0.001**	0.518	0.871	**0.002**	0.039	0.523	0.781
Na	**0.001**	0.310	0.891	0.153	0.482	0.623	0.478
P	**0.026**	0.889	0.323	**0.001**	**0.011**	0.959	0.381
16:0	**0.025**	0.256	**0.001**	**0.012**	0.741	0.330	0.957
18:0	0.173	0.855	**0.032**	0.672	0.636	**0.043**	**0.046**
SFA	0.006	0.877	**0.001**	0.315	0.858	**0.020**	**0.006**
18:1n − 9	0.059	0.876	0.101	0.959	0.527	0.305	**0.003**
MUFA	0.184	0.427	0.403	0.343	0.783	0.108	**0.001**
18:2n − 6	0.197	0.379	**0.010**	0.298	0.067	0.442	0.988
18:3n − 3	0.908	0.402	**0.048**	0.951	0.524	0.195	**0.014**
20:4n − 6	0.116	**0.015**	0.615	0.813	0.152	**0.033**	0.452
20:5n − 3	0.083	0.400	**0.001**	0.104	0.207	0.153	0.864
22:6n − 3	**0.001**	**0.001**	**0.001**	**0.001**	0.887	0.839	0.357
n − 6 PUFA	0.385	0.845	**0.001**	0.874	0.657	**0.398**	0.159
n − 3 PUFA	**0.015**	0.215	**0.002**	**0.001**	0.728	**0.051**	0.340
LC-PUFA	**0.029**	0.130	**0.001**	0.092	0.628	0.144	0.099
n − 3/n − 6	0.079	0.230	0.788	**0.009**	0.831	0.124	**0.044**
GPx	**0.006**	**0.023**	0.666	0.958	0.958	**0.001**	0.768
SOD	**0.039**	0.215	0.445	0.410	0.242	**0.001**	0.732
CAT	**0.033**	0.260	0.575	0.529	0.296	**0.001**	0.655
GSH	**0.059**	0.327	0.432	0.580	**0.001**	**0.001**	0.567
MDA	**0.005**	**0.035**	0.737	0.775	0.768	**0.004**	0.577
TP	0.106	0.286	0.989	0.666	0.829	0.188	0.820
PO	0.155	0.854	0.371	0.231	0.669	0.903	0.210
THC	0.494	0.781	0.381	0.384	0.781	0.781	0.242
GC	0.897	0.443	0.256	0.256	1.000	0.256	0.797
SGC	0.537	0.790	0.429	0.429	0.790	0.537	0.790
HC	0.706	0.499	0.499	0.499	0.820	0.410	0.598
*igf*	**0.001**	**0.001**	0.770	**0.001**	0.906	**0.001**	0.410
*lec*	**0.001**	**0.001**	0.981	**0.001**	**0.003**	0.471	**0.001**
*pen-3*	0.629	**0.001**	**0.002**	0.243	0.953	**0.011**	**0.029**
*propo*	0.206	**0.001**	0.663	**0.001**	0.129	**0.038**	**0.001**
*lyz*	**0.001**	**0.021**	**0.027**	0.709	0.979	0.714	**0.001**

*Note:* K, Fulton's condition factor. The significance of the two main effects (fish meal replacement level and acidifier level) and their interaction were analyzed using two-way ANOVA and significant values under *p* < 0.05 showed in bold.

Abbreviations: Ca, calcium; CAT, catalase; FBL, final body length; FBW, final body weight; FCR, feed conversion ratio; FI, feed intake; GC, granular cells; GPx, glutathione peroxidase; GSH, glutathione; HC, hyaline cells; HPI, hepatopancreatic index; IBL, initial body length; IBW, initial body weight; igf, insulin-like growth factor hormone; K, potassium; LC-PUFA, long-chain polyunsaturated fatty acids; lec, lectin; LPV, lipid productive value; lyz, lysozyme; MDA, malondialdehyde; Mg, magnesium; MUFA, monounsaturated fatty acids; Na, sodium; P, phosphorous; pen-3, peneidin-3; PO, phenoloxidase activity; PPV, protein productive value; propo, prophenoloxidase; PUFA, polyunsaturated fatty acids; Se, selenium; SFA, saturated fatty acids; SGC, semi granular cells; SGR, specific growth rate; SOD, superoxide dismutase; SUR, survival; THC, total hemocytes; TP, total protein; WG, weight gain.

^a^Experimental groups: FO_3_Se^0.4^SW (3%FO + 0.4 mg/kg SeNPs reared in SW), FO_3_Se^0.8^SW (3%FO + 0.8 mg/kg SeNPs reared in SW), FO_6_Se^0.4^SW (6%FO + 0.4 mg/kg SeNPs reared in SW), FO_6_Se^0.8^SW (6%FO + 0.8 mg/kg SeNPs reared in SW), FO_3_Se^0.4^HS (3%FO + 0.4 mg/kg SeNPs reared in HS), FO_3_Se^0.8^HS (3%FO + 0.8 mg/kg SeNPs reared in HS), FO_6_Se^0.4^HS (6%FO + 0.4 mg/kg SeNPs reared in HS), and FO_6_Se^0.8^HS (6%FO + 0.8 mg/kg SeNPs reared in HS).

**Table 6:** Proximate composition (%) of whole body and mineral content in *P. vannamei* fed various levels of FO and SeNP at various salinity for 56 days.

Parameters	Experimental groups^1^								
	FO_3_Se^0.4^SW	FO_3_Se^0.8^SW	FO_6_Se^0.4^SW	FO_6_Se^0.8^SW	FO_3_Se^0.4^HS	FO_3_Se^0.8^HS		FO_6_Se^0.4^HS	FO_6_Se^0.8^HS
Moisture	76.6 ± 0.7^b^	77.1 ± 0.8^a^	76.3 ± 0.5^b^	76.1 ± 0.1^b^	74.3 ± 0.4^d^		75.1 ± 0.4^c^	74.7 ± 0.4^d^	75.5 ± 0.4^c^
Crude protein	20.4 ± 0.5^ab^	19.8 ± 0.6^b^	20.5 ± 0.4^ab^	20.8 ± 0.1^ab^	22.0 ± 0.2^a^		21.4 ± 0.2^a^	21.7 ± 0.4^a^	21.0 ± 0.3^ab^
Crude lipid	0.6 ± 0.0	0.6 ± 0.0	0.6 ± 0.0	0.6 ± 0.0	0.7 ± 0.1		0.7 ± 0.0	0.6 ± 0.1	0.6 ± 0.0
Crude Ash	2.4 ± 0.1^b^	2.5 ± 0.2^b^	2.6 ± 0.2^b^	2.5 ± 0.0^b^	3.0 ± 0.1^a^		2.8 ± 0.1^a^	3.0 ± 0.1^a^	2.9 ± 0.1^a^
Minerals									
Se (mg/kg)	4.0 ± 0.3^b^	4.7 ± 0.2^a^	3.5 ± 0.2^c^	4.2 ± 0.3^ab^	4.2 ± 0.1^ab^		3.4 ± 0.3^c^	3.7 ± 0.1^c^	3.3 ± 0.1^c^
Ca (g/kg)	3.3 ± 0.1^a^	3.2 ± 0.2^a^	3.4 ± 0.1^a^	2.7 ± 0.1^b^	2.6 ± 0.1^c^		2.5 ± 0.0^c^	2.1 ± 0.1^d^	2.3 ± 0.0^d^
K (g/kg)	23.4 ± 1.3^b^	23.4 ± 1.7^b^	24.0 ± 0.5^b^	26.2 ± 0.2^a^	21.7 ± 0.7^c^		23.5 ± 0.4^b^	20.0 ± 0.6^d^	18.7 ± 0.4^d^
Mg (g/kg)	1.8 ± 0.1^b^	2.0 ± 0.1^ab^	2.0 ± 0.0^a^	2.1 ± 0.0^a^	1.9 ± 0.0^b^		1.8 ± 0.0^b^	1.7 ± 0.1^c^	1.5 ± 0.1^d^
P (g/kg)	7.2 ± 0.6^e^	7.8 ± 0.5^d^	8.2 ± 0.2^b^	9.2 ± 0.2^a^	8.1 ± 0.3^b^		8.1 ± 0.3^b^	7.2 ± 0.1^e^	6.6 ± 0.2^f^
Na (g/kg)	7.4 ± 0.3^a^	7.5 ± 0.3^a^	7.5 ± 0.2^a^	7.6 ± 0.2^a^	6.8 ± 0.1^b^		6.9 ± 0.2^b^	6.6 ± 0.4^c^	6.2 ± 0.3^d^

*Note:* Data are presented as the mean ± pooled SE of three replicates. Values within a column with a common superscript letter are not significantly different from the other dietary groups (*p* > 0.05).

Abbreviations: Ca, calcium; K, potassium; Mg, magnesium; Na, sodium; P, phosphorous; Se, selenium.

^1^Experimental groups: FO_3_Se^0.4^SW (3%FO + 0.4 mg/kg SeNPs reared in SW), FO_3_Se^0.8^SW (3%FO + 0.8 mg/kg SeNPs reared in SW), FO_6_Se^0.4^SW (6%FO + 0.4 mg/kg SeNPs reared in SW), FO_6_Se^0.8^SW (6%FO + 0.8 mg/kg SeNPs reared in SW), FO_3_Se^0.4^HS (3%FO + 0.4 mg/kg SeNPs reared in HS), FO_3_Se^0.8^HS (3%FO + 0.8 mg/kg SeNPs reared in HS), FO_6_Se^0.4^HS (6%FO + 0.4 mg/kg SeNPs reared in HS), and FO_6_Se^0.8^HS (6%FO + 0.8 mg/kg SeNPs reared in HS).

**Table 7:** Fatty acid profile (%) of whole body in *P. vannamei* fed various levels of FO and SeNP at various salinity for 56 days.

Parameters	Initial	Experimental groups^1^								
		FO_3_Se^0.4^SW	FO_3_Se^0.8^SW	FO_6_Se^0.4^SW	FO_6_Se^0.8^SW	FO_3_Se^0.4^HS	FO_3_Se^0.8^HS		FO_6_Se^0.4^HS	FO_6_Se^0.8^HS
16:0	17.0	19.4 ± 0.7^b^	21.5 ± 0.9^ab^	19.1 ± 0.5^b^	20.0 ± 0.5^ab^	19.5 ± 1.0^b^		21.1 ± 0.7^ab^	22.2 ± 1.0^a^	22.8 ± 0.7^a^
18:0	8.3	14.1 ± 1.5^c^	23.5 ± 1.9^a^	18.8 ± 1.9^b^	18.2 ± 1.1^b^	17.2 ± 1.1^b^		22.7 ± 2.2^a^	17.9 ± 0.6^b^	23.4 ± 1.5^a^
SFA^2^	27.4	37.6 ± 1.4^c^	52.2 ± 1.5^a^	43.5 ± 1.5^b^	43.1 ± 1.5^b^	44.7 ± 2.7^b^		50.6 ± 3.1^a^	45.2 ± 0.7^b^	52.5 ± 1.1^a^
18:1n-9	22.1	23.0 ± 0.6^a^	18.1 ± 0.9^b^	19.2 ± 1.2^b^	22.2 ± 1.4^a^	18.8 ± 1.9^b^		18.9 ± 1.4^b^	21.0 ± 1.0^ab^	18.9 ± 1.0^b^
MUFA^3^	26.2	28.8 ± 0.2^a^	23.4 ± 0.2^b^	22.4 ± 1.3^b^	26.8 ± 1.3^ab^	23.6 ± 1.5^b^		24.8 ± 1.2^ab^	25.8 ± 1.6^ab^	22.8 ± 1.2^b^
18:2n − 6	21.3	12.4 ± 0.4^a^	7.5 ± 0.3^b^	12.0 ± 0.5^a^	8.2 ± 0.6^b^	10.8 ± 0.9^ab^		8.7 ± 1.3^b^	8.9 ± 1.5^b^	7.8 ± 1.4^b^
18:3n − 3	1.4	0.5 ± 0.2^c^	2.3 ± 0.8^ab^	1.4 ± 0.1^b^	2.1 ± 0.5^ab^	2.0 ± 0.4^ab^		0.9 ± 0.2^c^	0.6 ± 0.2^c^	3.0 ± 0.9^a^
20:4n − 6 (ARA)	3.6	2.2 ± 0.3^ab^	1.7 ± 0.1^b^	2.2 ± 0.5^ab^	3.1 ± 0.4^a^	2.1 ± 0.2^ab^		1.2 ± 0.2^b^	2.3 ± 0.5^ab^	2.2 ± 0.3^ab^
20:5n − 3 (EPA)	6.7	6.6 ± 0.8^a^	3.2 ± 0.3^b^	6.4 ± 0.6^a^	4.2 ± 0.6^ab^	6.0 ± 0.8^a^		3.7 ± 0.7^b^	3.9 ± 0.3^b^	3.2 ± 0.9^b^
22:6n − 3 (DHA)	10.1	6.2 ± 0.7^ab^	4.4 ± 0.5^b^	8.7 ± 0.1^a^	7.3 ± 0.2^a^	6.1 ± 0.1^ab^		4.9 ± 0.5^b^	6.2 ± 0.4^ab^	4.4 ± 0.3^b^
n − 6 PUFA^4^	27.2	18.9 ± 0.3^a^	13.4 ± 0.5^c^	17.1 ± 0.7^ab^	15.3 ± 0.2^b^	16.6 ± 1.6^b^		14.1 ± 1.1^c^	17.3 ± 1.3^ab^	13.9 ± 1.6^c^
n − 3 PUFA	19.1	14.5 ± 1.5^ab^	10.6 ± 0.7^b^	16.9 ± 0.4^a^	14.7 ± 1.1^ab^	15.3 ± 0.4^a^		10.4 ± 1.5^b^	11.6 ± 0.6^b^	11.4 ± 1.3^b^
LC-PUFA^5^	23.4	20.2 ± 1.6^a^	13.9 ± 0.8^b^	20.3 ± 0.9^a^	19.4 ± 1.6^a^	18.7 ± 1.2^a^		14.4 ± 1.1^b^	18.7 ± 1.2^a^	14.1 ± 0.9^b^
n − 3/n-6	0.7	0.8 ± 0.1^b^	0.8 ± 0.1^b^	1.0 ± 0.1^a^	1.0 ± 0.1^a^	0.9 ± 0.1^ab^		0.7 ± 0.1^b^	0.7 ± 0.0^b^	0.8 ± 0.1^b^
DHA/EPA	1.5	0.9 ± 0.1^c^	1.4 ± 0.2^b^	1.4 ± 0.2^b^	1.8 ± 0.3^a^	1.0 ± 0.1^c^		1.4 ± 0.2^b^	1.6 ± 0.2^b^	1.4 ± 0.7^b^
ARA/EPA	0.5	0.3 ± 0.1^b^	0.6 ± 0.1^ab^	0.3 ± 0.1^b^	0.8 ± 0.1^a^	0.4 ± 0.1^b^		0.4 ± 0.1^b^	0.6 ± 0.2^ab^	0.7 ± 0.3^a^

*Note:* Data are presented as the mean ± pooled SE of three replicates. Values within a column with a common superscript letter are not significantly different from the other dietary groups (*p* > 0.05).

^1^Experimental groups: FO_3_Se^0.4^SW (3%FO + 0.4 mg/kg SeNPs reared in SW), FO_3_Se^0.8^SW (3%FO + 0.8 mg/kg SeNPs reared in SW), FO_6_Se^0.4^SW (6%FO + 0.4 mg/kg SeNPs reared in SW), FO_6_Se^0.8^SW (6%FO + 0.8 mg/kg SeNPs reared in SW), FO_3_Se^0.4^HS (3%FO + 0.4 mg/kg SeNPs reared in HS), FO_3_Se^0.8^HS (3%FO + 0.8 mg/kg SeNPs reared in HS), FO_6_Se^0.4^HS (6%FO + 0.4 mg/kg SeNPs reared in HS), and FO_6_Se^0.8^HS (6%FO + 0.8 mg/kg SeNPs reared in HS).

^2^SFA includes: 14:0, 15:0, 17:0, and 20:0.

^3^MUFA includes: 14n − 1, 16n − 7, 18n − 7, and 20:1n − 4.

^4^PUFA includes: 20:3n − 6, 22:4n − 6, 22:5n − 6, and 22:5n − 3.

^5^LC-PUFA includes: 20:3n − 6, 20:4n − 6, 20:5n − 3, 22:4n − 6, 22:5n − 6, 22:5n − 3, and 22:6n − 3.

**Table 8:** Hemato-immunological parameters of *P. vannamei* juveniles fed various levels of FO and SeNP at various salinity for 56 days.

Parameters	Experimental groups^a^								
	FO_3_Se^0.4^SW	FO_3_Se^0.8^SW	FO_6_Se^0.4^SW	FO_6_Se^0.8^SW	FO_3_Se^0.4^HS		FO_3_Se^0.8^HS	FO_6_Se^0.4^HS	FO_6_Se^0.8^HS
TP (g/dL)	5.9 ± 0.5	4.8 ± 0.7	4.6 ± 1.4	5.4 ± 0.9	6.8 ± 0.7	6.3 ± 1.0		5.3 ± 0.3	6.1 ± 0.5
PO (U/mL)	0.5 ± 0.0	0.5 ± 0.0	0.5 ± 0.0	0.5 ± 0.0	0.6 ± 0.0	0.5 ± 0.0		0.6 ± 0.0	0.6 ± 0.0
THC (×10^6^ cell/mL)	8.0 ± 1.2	8.2 ± 1.4	8.6 ± 0.8	6.8 ± 0.6	8.5 ± 0.6	7.4 ± 0.6		8.7 ± 1.1	8.1 ± 0.3
GC (%)	29.0 ± 2.1	29.0 ± 2.0	28.3 ± 2.8	24.7 ± 0.7	27.6 ± 2.0	27.0 ± 1.0		29.3 ± 0.7	26.3 ± 1.9
SGC (%)	44.7 ± 1.7	44.3 ± 0.9	45.3 ± 1.2	45.7 ± 1.2	46.0 ± 1.0	45.7 ± 1.3		44.7 ± 1.8	46.0 ± 1.2
HC (%)	26.3 ± 1.5	26.7 ± 1.5	26.3 ± 1.7	29.7 ± 0.9	26.3 ± 1.2	27.3 ± 2.3		26.0 ± 2.3	27.7 ± 2.0

*Note:* Data are presented as the mean ± pooled SE of three replicates.

^a^Experimental groups: FO_3_Se^0.4^SW (3%FO + 0.4 mg/kg SeNPs reared in SW), FO_3_Se^0.8^SW (3%FO + 0.8 mg/kg SeNPs reared in SW), FO_6_Se^0.4^SW (6%FO + 0.4 mg/kg SeNPs reared in SW), FO_6_Se^0.8^SW (6%FO + 0.8 mg/kg SeNPs reared in SW), FO_3_Se^0.4^HS (3%FO + 0.4 mg/kg SeNPs reared in HS), FO_3_Se^0.8^HS (3%FO + 0.8 mg/kg SeNPs reared in HS), FO_6_Se^0.4^HS (6%FO + 0.4 mg/kg SeNPs reared in HS), and FO_6_Se^0.8^HS (6%FO + 0.8 mg/kg SeNPs reared in HS).

## Data Availability

The datasets generated and/or analyzed during the current study are available from the corresponding author upon reasonable request.

## Supporting Information

Supporting Information Figure S1. Supplementing diet with 6% fish oil and 0.4 mg/kg selenium nanoparticles improved growth, antioxidant capacity, and immune responses in Penaeus vannamei juveniles reared in hypersaline water (50 g/L).
