# Supplementary material for: The Interactive Effects of Dietary Fish Oil and Selenium Nanoparticles Increased Growth, Antioxidant Capacity, and Immune-Related Genes Transcription Level in Penaeus vannamei Reared in Hypersaline Water
Source: Aquac Nutr. 2025 Aug 28;2025:4165191. doi: 10.1155/anu/4165191 (PMC12411063; doi:10.1155/anu/4165191)
Supplement: Supporting Information — Figure S1. Supplementing diet with 6% fish oil and 0.4 mg/kg selenium nanoparticles improved growth, antioxidant capacity, and immune responses in Penaeus vannamei juveniles reared in hypersaline water (50 g/L). [file 4165191.f1.pptx]

## Slide 1
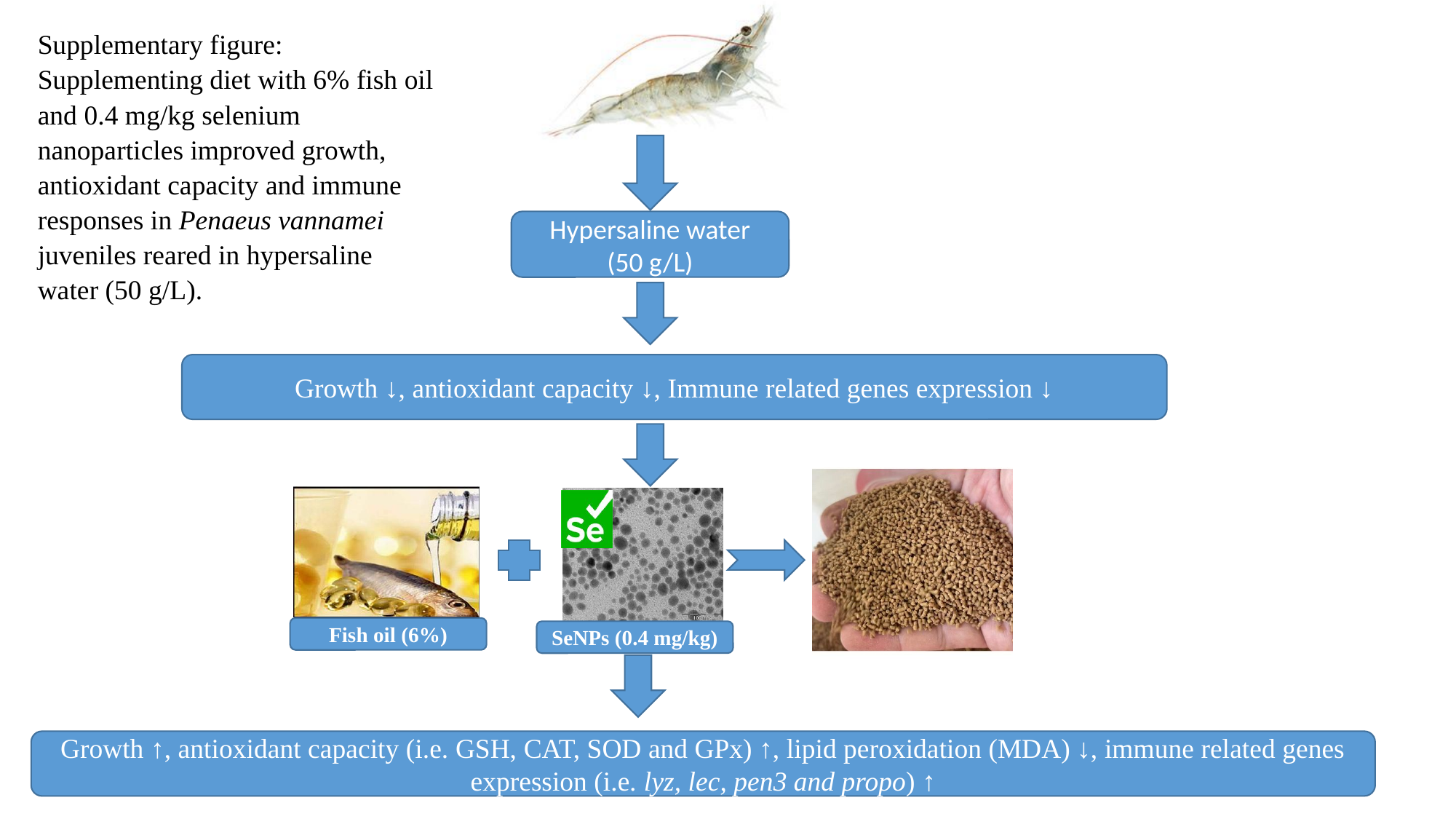

Supplementary figure: Supplementing diet with 6% fish oil and 0.4 mg/kg selenium nanoparticles improved growth, antioxidant capacity and immune responses in Penaeus vannamei juveniles reared in hypersaline water (50 g/L).
#
Hypersaline water
(50 g/L)
Growth ↓, antioxidant capacity ↓, Immune related genes expression ↓
Fish oil (6%)
SeNPs (0.4 mg/kg)
Growth ↑, antioxidant capacity (i.e. GSH, CAT, SOD and GPx) ↑, lipid peroxidation (MDA) ↓, immune related genes expression (i.e. lyz, lec, pen3 and propo) ↑
